# Impacts of Biological Heating and Degradation during Bale Storage on the Surface Properties of Corn Stover

**DOI:** 10.1021/acssuschemeng.0c03356

**Published:** 2020-08-13

**Authors:** Elizabeth Bose, Juan H. Leal, Amber N. Hoover, Yining Zeng, Chenlin Li, Allison E. Ray, Troy A. Semelsberger, Bryon S. Donohoe

**Affiliations:** † Bioenergy Center, National Renewable Energy Laboratory (NREL), 15013 Denver West Parkway, Golden, Colorado 80401, United States; ‡ Material Physics Applications Division, Los Alamos National Laboratory, P.O. Box 1663, Los Alamos, New Mexico 87545, United States; § Energy & Environment Science & Technology, Idaho National Laboratory, 1955 N. Fremont Avenue, Idaho Falls, Idaho 83415, United States

**Keywords:** biomass feedstock, surface characterization, corn stover, biological degradation, surface area, porosity

## Abstract

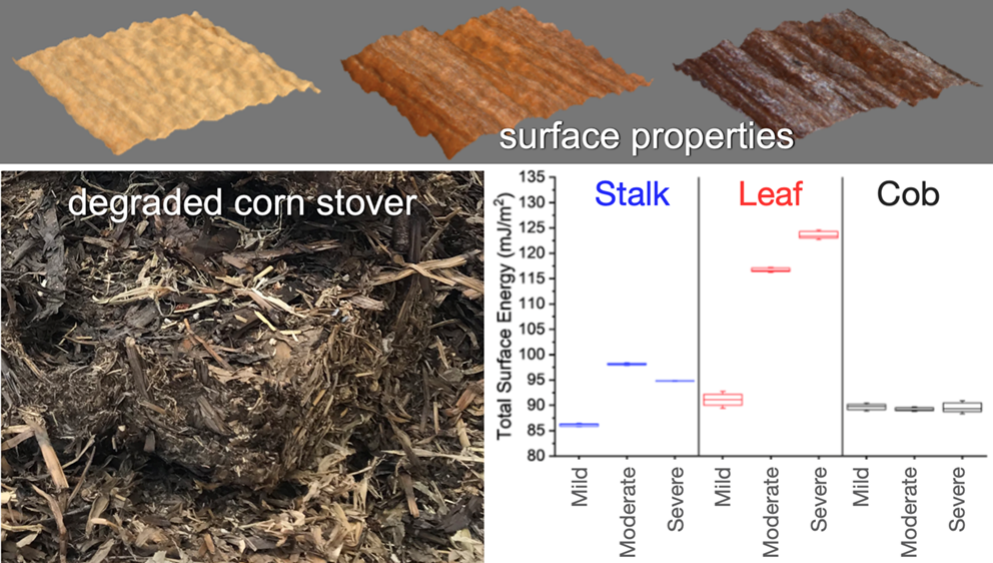

The variability of chemical, physical, and mechanical properties of lignocellulosic biomass feedstocks has a major impact on the efficiency of biomass processing and conversion to fuels and chemicals. Storage conditions represent a key source of variability that may contribute to biomass quality variations from the time of harvest until delivery to the biorefinery. In some cases, substantial microbial degradation can take place during storage. In this work, we investigate how degradation during storage affects the surface texture, surface energy, and porosity of different corn stover anatomical fractions (e.g., leaf, stalk, and cob). Understanding any potential changes in surface properties is important because interparticle interactions during bioprocessing cause aggregation and blockages that lead to at least process inefficiency and at most complete equipment failure. The surface roughness and texture parameters of corn stover with variable degrees of microbial degradation were calculated directly from stereomicroscopy and scanning electron microscopy micrographs. Surface energy and porosity were measured by inverse gas chromatography. The results show differing trends in the impact of increasing biological heating and degradation depending on the specific corn stover tissue type that was analyzed. These results also indicate that biomass surface properties are scale-dependent and that the scale, which is most industrially relevant, may depend on the specific unit operation within the biorefinery being considered.

## Abstract 2

Fundamental understanding of variability in biomass surface properties across multiple scales informs development of advanced processing technologies to improve feedstock handling for sustainable biomass conversion.

## Introduction

The demand for alternative, sustainable replacements for petroleum-based fuels and platform chemicals is driven by high energy demands, concern over global climate change, and the unstable geopolitics of petroleum production.^1−3^ Lignocellulosic biomass is a promising alternative resource to displace some of the supply because it is abundant and renewable and its use can boost local U.S. economies.^2−5^

In the U.S., one of the most abundant and readily available sources of lignocellulosic biomass is the agricultural residue, corn (*Zea mays*) stover.^6^ Corn stover will be an important resource for the commercial and economic viability of many integrated biorefineries (IBRs) where lignocellulosic biomass is converted into biofuel and other bioproducts. Successfully shifting production of petroleum-based products to bioproducts depends on economic efficiency of integrated biorefineries, but only a few commercial-scale IBRs are currently operating.^7^ One of the major barriers impeding the growth of IBR commercialization is the challenge of handling bulk biomass feedstocks. The fibrous, irregular, and variable shape and surface properties of milled lignocellulosic biomass promote agglomeration, plugging, and arching in equipment, contributing to inefficient processing and excessive downtime for feedstock handling equipment.^7,8^ A suspected underlying cause of these technical challenges is broad variability in feedstock properties.^9,10^

Feedstock variability is a general term for the distribution in thermophysical and chemical properties of harvested biomass.^8^ Generally, more attention has been paid to the essential issues of organic and inorganic compositional variability that impact conversion yields than to the emergent critical properties^11^ at the micro and millimeter scales, which directly impact biomass conveyance and particle flow.^9,12,13^

**Figure 1:**
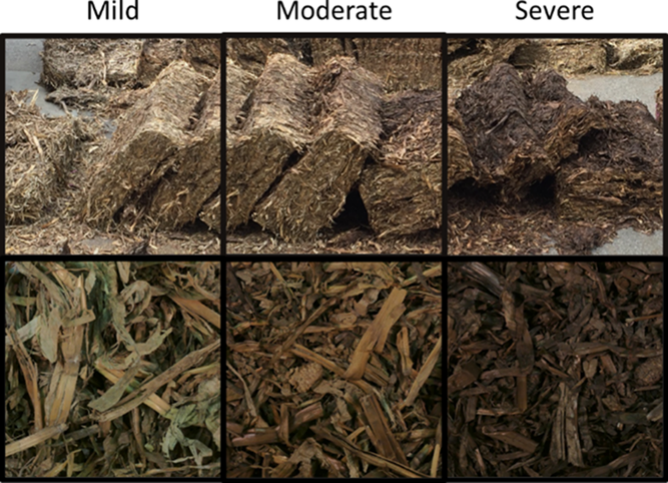
Biologically heated bale variability (upper panels) and samples taken from each visually identified degradation state within the bale—mild, moderate, and severe (lower panels). Figure modified from images previously published by Groenewold et al.^26^ and Li et al.^27^

One major source of the variability is storage conditions,^14^ which is fundamental to the supply chain, given the seasonal harvest window of biomass, in contrast to biorefineries that require a continuous, year-long feedstock supply.^15−17^ Among the challenges of biomass in field storage is that degradation can occur, although biological heating, also referred to as self-heating,^18^ occurs in piles^19^ or bales of the organic material like corn stover^15,20,21^ (Figure 1). It occurs as a result of continued respiration of the plant tissues, abiotic oxidation, and microbial degradation, and a raised temperature that can reach up to 80 °C^20^ and can cause dry matter loss and reduced conversion yields results in the loss of valuable sugars.^22^ Biological degradation can occur after baling as a result of factors like intrabale moisture content, oxygen conditions in the bale, location in the field-side stack, and microbial communities present. High moisture stimulates microbial activity within bales, generating heat, leading to loss of moisture and physicochemical alterations in biomass, and has been demonstrated in tightly controlled conditions at the laboratory scale with storage simulators^23^ and in the field.^24^

Biological heating is an exothermic aerobic reaction that continues to increase bale temperature until spontaneous oxidation takes over. The effects of biological heating can be grouped into three mechanistic categories—biological degradation, thermal degradation, and partial/complete oxidation.^25^ Biological degradation is a set of selective microbial enzyme reactions that target specific chemical linkages. Thermal degradation is a consequence of increasing temperature that can take place with or without the presence of oxygen. The extent of thermal degradation is directly related to the temperature gradient within the bale. The continued heating of the bale raises the temperature to a point where oxidative degradation starts.^20^ Complete oxidation of corn stover can only proceed if the supply of oxygen is sufficient for complete combustion.^25^ Prior to complete combustion, the oxygen supply for complete combustion is limited (e.g., diffusion limited), and therefore, oxidation of corn stover is limited to partial oxidation.^20^ The partial oxidation product spectrum is a function of the temperature, time, and oxygen supply.

Understanding how biological heating contributes to compositional, interfacial, and structural variations will provide a clearer picture of the breadth of feedstock variability so that processes can be better engineered and managed to accommodate a material with a range of thermal and moisture histories due to storage and still promote continuous operation. In addition to the chemical changes that take place, an important variable aspect of biologically heated feedstock is the surface microstructure, which can also be referred to as the surface texture. The surface texture of a material is industrially relevant because it can cause higher interparticle friction forces that result in poor feeding^28^ and flowability.^8,9,29^ Higher surface roughness is also correlated with hydrophobicity;^30^ thus, rougher materials may be more resistant to wetting by aqueous catalyst solutions during conversion. Conversely, feedstock with smoother surfaces will likely exhibit better flow behavior that may minimize storage and transportation volumes, reduce stress on storage structures, facilitate mixing and blending, enable better feeding, and allow more efficient emptying and cleaning of equipment.^9^ Hydrophobicity is also a component of surface energy. Surface energy is an emergent material property^11,31^ that is of interest because it is recognized as an important material attribute in other industries where bulk solid flow is critical to the process, and it is starting to be understood as a critical attribute for biomass.^8^ This study aims to characterize the variability of surface texture and surface energy parameters and compare them among corn stover samples and anatomical fractions that have been variably degraded through biological heating during field-side bale storage. This fundamental understanding of variable corn stover surface properties will inform the design and operation of processing and conversion operations.

## Materials and Methods

### Corn Stover Sample Acquisition, Sampling, and Anatomical Fraction Distribution

Corn stover was harvested and baled on October 27, 2017 in Story County, Iowa. The month prior to harvesting/baling had abundant precipitation (162 mm) and an average maximum daily temperature of 20 °C (https://www.ncdc.noaa.gov/cdo-web/, Station: USW00094989, Ames Municipal Airport, IA, US). Bales were then stored field-side where 12 mm of precipitation fell (average maximum daily temperature of 8 °C) until being transported and placed in covered storage at the Iowa State University on December 21, 2017. Average maximum daily temperature during this storage period was 0 °C. Bales were finally shipped to the Idaho National Laboratory, Idaho Falls, Idaho, and placed in covered storage on March 22, 2018. Evidence of bale degradation as dark brown/black areas on the outside of the bale was observed for this specific bale on March 23, 2018. Samples for this study were collected on April 24, 2019. The bale was dissected, and samples were collected and visually identified as mildly biologically heated (mild), moderately biologically heated (moderate), and severely biologically heated (severe) (Figure 1).

**Figure 2:**
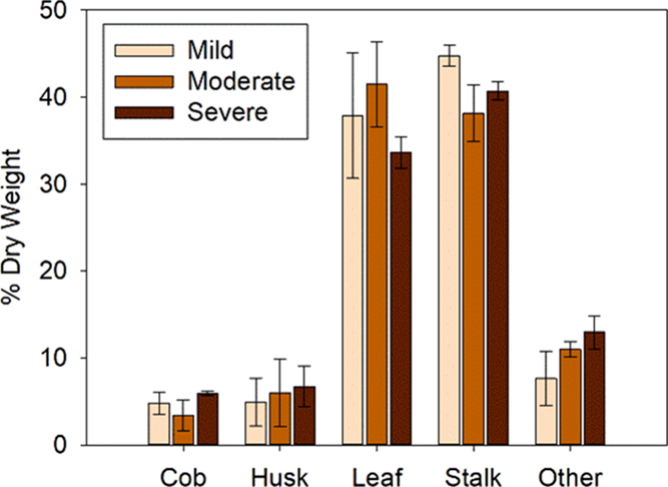
Percent weight on a dry basis of the primary anatomical fractions in the mildly, moderately, and severely biologically heated samples dissected from a corn stover bale (mean ± SD, *n* = 3). The “other” group includes minor fractions like tassels and materials that could not be identified (e.g., fines).

Three grab samples were taken from each biological heating level—mild, moderate, and severe. Dry weight (105 °C) of grab samples fractioned was 309 ± 66 g (mean ± SD, *n* = 9). Each sample was hand fractioned into cob, husk, leaf, stalk, and not identifiable/other, which included minor fractions like tassels. Weight of each fraction was determined after drying at 105 °C, and the percent of each fraction by dry weight was calculated. Leaves and stalk were dominant fractions by mass, accounting for an average of 74–83% of the stover for the degraded bale samples (Figure 2). Leaves and stalks were therefore emphasized for subsequent analyses.

### Sample Preparation for Imaging

Samples were prepared for image acquisition by selecting leaves and stalk pieces for analysis and cutting them to fit onto SEM stubs (15–30 mm squares). The leaf and stalk fractions were further divided and oriented as either leaf top, leaf bottom, stalk exterior, or stalk interior so that there were 10 samples of each for the mildly, moderately, and severely degraded group as well as the control. The samples were mounted onto aluminum stubs using double sided adhesive. The samples were sputter coated with 12 nm iridium using a Cressington sputter coater 208 HR (Cressington Scientific Instruments, Ltd., Watford, UK). The sputter coating was to prevent charging during SEM imaging and was used to normalize the differences in color among the three groups as the mild group was light tan while the moderate and severe groups were darker brown. This was to minimize the possibility that textural differences found would be influenced by color differences.

### Stereoscope and SEM Image Acquisition

Focal plane z-stacks of the samples were captured using a Nikon SMZ1500 stereomicroscope and a Nikon DS-Fi1 CCD camera that was operated with the Nikon Digital Sight system (Nikon Instruments, Melville, NY, USA). All images were taken under bright-field lighting with a fully open aperture. A Prior OptiScan III joystick (Prior Scientific Instruments Ltd., Fulbourn, Cambridge, UK) was used to change the focus in incremental steps (*z* = 3) so that the height difference between each image in the z-stacks was consistent among samples. The shutter speed was 1/10 s, and the magnification of the variable power objective was 2×.

Scanning electron microscopy micrographs of stalk and leaf were acquired with an FEI Quanta 400 FEG instrument (FEI, Hillsboro, OR, USA) under low vacuum at an accelerating voltage of 15 keV and using a gaseous solid-state detector (GAD) collecting secondary electrons. Corn cobs were imaged using a JEOL-JSM 6610LV scanning electron microscope (JEOL, Peabody, MA, USA) at 20 keV under low vacuum conditions and with a secondary electron detector.

### Stereoscope Image Processing

Fiji (https://fiji.sc) was used to convert the stereomicroscopy image z-stacks into depth maps.^32^ The image stacks were aligned using the Linear Stack Alignment with SIFT plugin. The EDF plugin^33^ was used with the EDF easy option and speed/quality set to the max and height-map reg set to the max with the default option to compute and generate the height map. Next, the Anaglyph plugin written by Dr. Gabriel Landini (School of Dentistry, University of Birmingham, UK) was used with the distance map option chosen to generate a depth map image that is the composite of the height map and output image of the EDF plugin.

### Surface Textural Analysis

Texture analysis was also performed using Fiji/ImageJ. The plugin SurfCharJ 1q^34^ was used, and its calculated root-mean-square deviation (Rq) and topographical surface area were compared. The plugin FracLac (imagej.nih.gov/ij/plugins/fraclac/FLHelp/Introduction.htm) was used to calculate fractal dimensions of the images. The Gray 2: different volume variation and block scan options were used.

### Statistical Analysis of Surface Texture

Statistical analysis on textural features was performed using R statistical software (https://www.r-project.org). Nonparametric tests were used to analyze the textural features of interest after visualization of histograms made non-normality of the sample distributions apparent. A Kruskal–Wallis rank sum test was used to determine if significant differences in values existed within anatomical fraction groups. A value of *P* < 0.05 was considered to be significant. The Dunn test^35^ was the post-hoc test used to determine which conditions were significantly different from each other within anatomical fraction groups. The Bonferroni method was used to correct *p* values. The Kruskal–Wallis test determines if samples are from the same distribution.^36^ The null hypothesis of the Dunn test is that the probability of observing a randomly selected value from the first group that is larger than a randomly selected value from the second group is one half; thus, it can be understood as a test for the difference between the medians (cran.r-project.org/web/packages/dunn.test/index.html. A value of *P* < 0.025 was considered to be significant, and a value of *p* < 0.10 was considered to be marginally significant.

### BET Porosity and Surface Area

A Micromeritics 3Flex instrument was used to collect the multipoint Brunauer–Emmett–Teller (BET) specific surface area (SSA). Nitrogen was used as the adsorbate for all BET analyses. All anatomical corn stover samples used for surface area, porosity, and surface energy analyses were milled to 2 mm. After preliminary bulk drying, at 45 °C for a minimum of 24 h using our in-house built argon-purged drying station, the samples, ranging in mass from 1.3 to 1.6 g, were further conditioned under vacuum at 45 °C until isobaric conditions were reached (constant, ≤1 ×10^–5^ mmHg) to ensure that the most accurate, reliable, and consistent surface areas were collected. The typical mass loss before and after the final conditioning step for all samples was less than 5 × 10^–4^ g. At a minimum, all samples were performed in triplicate using different samples. The standard error observed for the instrument using an NIST calibration reference (BCR-170, SA = 1.05 m^2^/g) was below 0.05 m^2^/g.

### Surface Energy by IGC (Inverse Gas Chromatography)

Surface energy measurements were carried out at infinite dilution using a surface energy analyzer (SEA) from Surface Measurement Systems, outfitted with a flame ionization detector (FID). Dry corn stover samples were packed in silanized glass columns using the same samples as those used for surface area measurements. All measurements were performed, at a minimum, in triplicate. Dispersive surface energy (γ_s_^d^) was estimated using HPLC grade *n*-alkanes (C_7_–C_10_) from Sigma-Aldrich. A monopolar Lewis acid and base, trichloromethane and ethyl acetate, respectively, of HPLC grade were procured from Sigma-Aldrich for the specific surface energy (γ_s_^ab^) estimations. Column packing densities ranged from 0.13 to 0.64 g/cm^3^, depending on the anatomical fraction. The dimensions of the silanized glass columns were maintained at 4 mm ID and 6 mm OD × 300 mm *L* for all analyses. Surface energy measurements were performed at infinite dilution (0.005 *n*/*n*_m_ or 0.5% monolayer coverage) and 30 °C and with a helium carrier gas at 10 sccm. The dispersive surface energy component was calculated using the Dorris–Gray method, and the acid–base (or specific) surface energy components were calculated using the van Oss–Chaudhury–Good (vOCG) polarization method. Instrument reproducibility is within 0.5% deviation using the mannitol reference standard provided by Surface Measurement Systems.

## Results and Discussion

### Topographical Surface Texture Measured by Image Analysis Reveals Different Trends with Biological Degradation Depending on the Tissue Type (Leaf vs Stalk) and Scale

**Figure 3:**
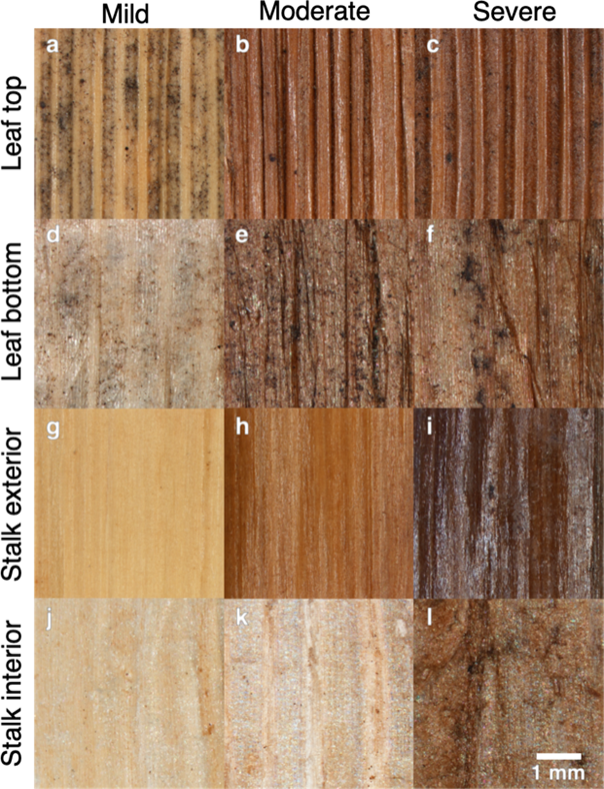
Stereomicrographs show that the texture and color of each sample vary with the amount of degradation and the anatomical fraction.( a–c) Mildly, moderately, and severely degraded leaf top, respectively. (d–f) Mildly, moderately, and severely degraded leaf bottom, respectively. (g–i) Mildly, moderately, and severely degraded stalk exterior, respectively. (j–l) Mildly, moderately, and severely degraded stalk interior, respectively. Scale bar = 1 mm.

**Figure 4:**
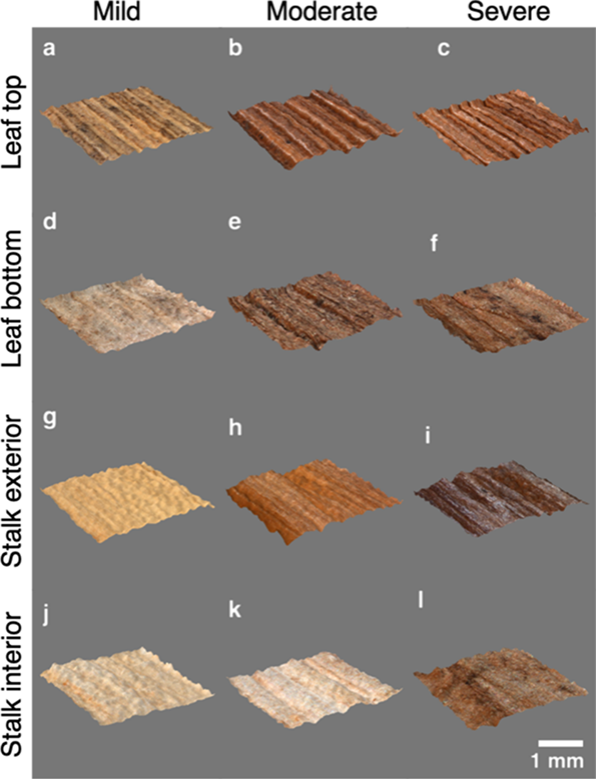
3D topology views generated from the EDF plugin in Fiji applied to the image stacks show the surface variation within and among the different anatomical fractions and degrees of biological heating severity. (a–c) Mildly, moderately, and severely degraded leaf top, respectively. (d–f) Mildly, moderately, and severely degraded leaf bottom, respectively. (g–i) Mildly, moderately, and severely degraded stalk exterior, respectively. (j–l) Mildly, moderately, and severely degraded stalk interior, respectively. Scale bar = 1 mm.

Ten samples of each anatomical fraction per sample group were chosen to best represent the variability of the sample. The stereomicroscopy output images (Figure 3) illustrate the color changes that occur from biological heating. Corn stover becomes progressively darker with increasing biological heating severity. Color was used during the initial sample selection and classification to determine the amount of degradation that samples underwent. Samples with limited degradation based on their light tan color were assigned to the mildly degraded group, and samples that had undergone the most degradation, as determined by their dark brown color, were assigned to the severely degraded group. Samples that were light brown were assigned to the moderately degraded group. One interesting observation about the color changes on the various corn stove tissues is that the moderately degraded stalk interior (Figure 3k) did not look different than the mild sample. This suggests that mild and moderate degradation may be limited to the surface and only in the most severe cases does the degradation penetrate deep into the biomass samples. The surface topology view images (Figure 4) show the three-dimensional surface texture at the millimeter scale. Texture is a variable among the leaf top, leaf bottom, stalk exterior, and stalk interior groups. The amount of variation among the three levels of degradation for each anatomical fraction is harder to differentiate by visual inspection and was quantified by image processing.

**Figure 5:**
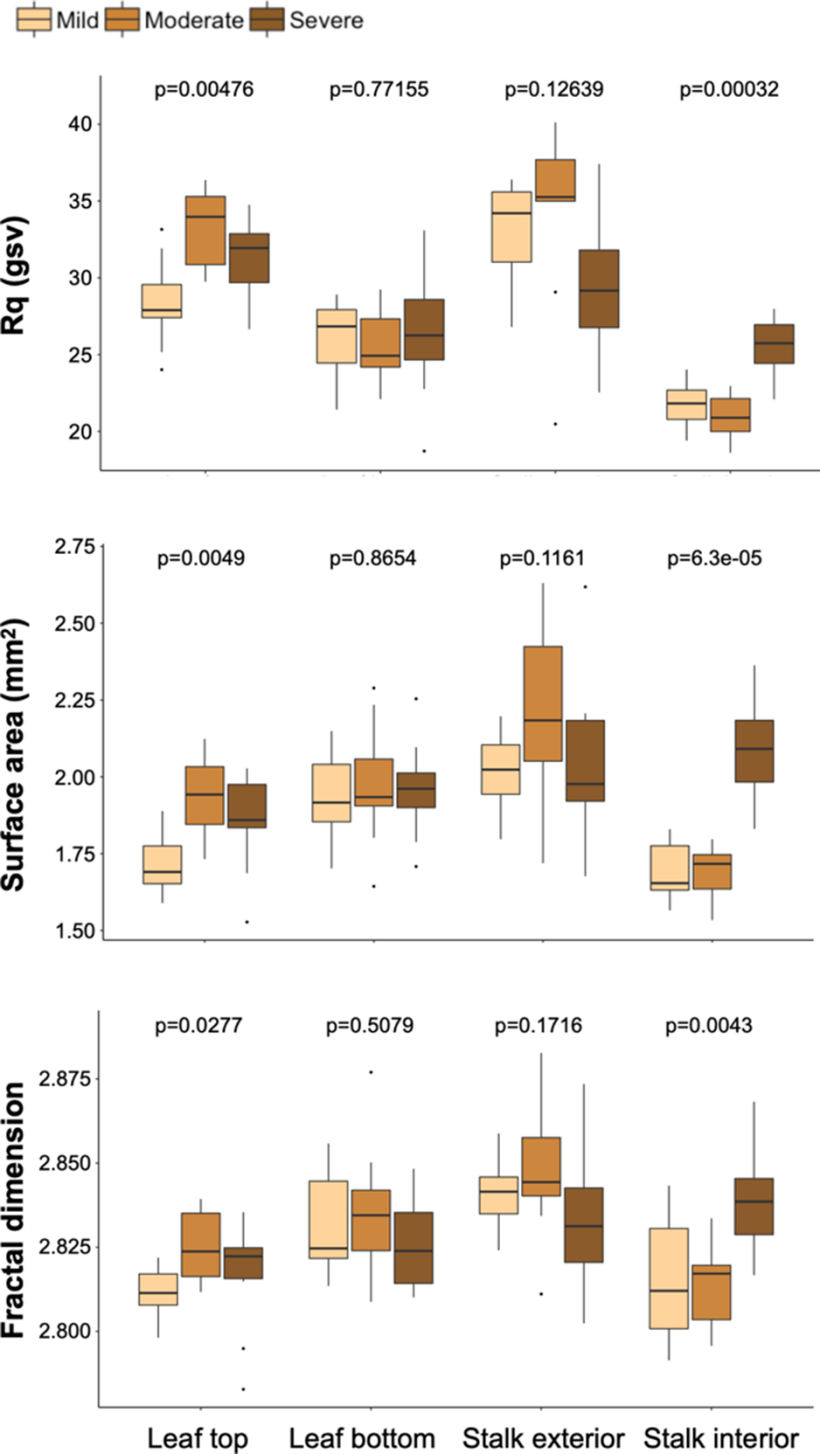
Surface texture quantification of stereoscopy images. The Rq, fractal dimension, and topographical surface area were assessed. A Kruskal–Wallis test was used to assess significant differences among the varying levels of degradation for each anatomical fraction, and the Dunn test was used for post-hoc analysis. The Rq, fractal dimension, and topographical surface area varied significantly within the leaf top and stalk interior groups such that increased biological degradation is related to higher surface roughness and surface area at the millimeter scale.

Use of imaging to quantify the surface texture was chosen because it matches the spatial scales of particles and surfaces of industrial relevance to biomass processing, and it is a nondestructive and relatively inexpensive characterization technique that could eventually be employed by industry. While the plugins used (SurfCharJ 1q and FracLac) calculated a total of 16 different textural parameters, Rq, topographical surface area, and fractal dimension were compared because they are most clearly related to particle behaviors of interest (Figure 5). The Rq of the samples was evaluated because Rq is a commonly used measure of surface roughness.^37^ It is the root-mean-square deviation from the mean line of the sample profile. When assessed via imaging using the SurfCharJ 1q plugin, Rq is calculated based on the grayscale values (gsv). The Rq values varied significantly within the leaf top and stalk interior groups (Kruskal–Wallis rank sum test; leaf top: *X*^2^ = 10.694, df = 2, and *p* value < 0.001; stalk interior: *X*^2^ = 16.114, df = 2, and *p* value < 0.001; Table S2. The moderately degraded leaf tops had a significantly higher distribution of Rq values than the mildly degraded leaf tops (Dunn’s test; *z* = −3.25 and *p* < 0.01), and the severely degraded stalk interior surfaces had a significantly higher distribution of Rq values than the mildly and moderately degraded stalk interior surfaces (Dunn’s test; mild–severe: *z* = −3.10 and *p* < 0.01; moderate–severe: *z* = −3.76 and *p* < 0.001). The severely degraded leaf tops had a marginally higher distribution of Rq values than the mildly degraded leaf tops (Dunn’s test; *z* = −1.93 and *p* = 0.0803). These results indicate that increased biological degradation is associated with rougher leaf top and stalk interior surfaces. This higher surface roughness may lead to more interparticle friction and poorer flowability.

The topographical surface area varied significantly within the leaf top and stalk interior groups (Kruskal–Wallis rank sum test; leaf top: *X*^2^ = 10.64, df = 2, and *p* value < 0.01; stalk interior: *X*^2^ = 19.36, df = 2, and *p* value < 0.001; Table S2). The moderately degraded leaf tops had a higher topographical surface area distribution than the mildly degraded leaf tops (Dunn’s test; *z* = −3.18 and *p* < 0.01), and the severely degraded leaf tops had a marginally significantly higher surface area than the mildly degraded leaf tops (Dunn’s test; *z* = −2.24 and *p* = 0.0381). The severely degraded stalk interior topographical surface area is significantly higher than the mildly and moderately degraded stalk interior groups (Dunn’s test; mild–severe: *z* = −3.81 and *p* < 0.001; moderate–severe: *z* = −3.81 and *p* < 0.001).

Fractal analysis was also conducted because the fractal dimension (*D*_f_) of a surface is an indicator of its roughness and it is positively correlated with higher static friction between surface interfaces.^38^ The fractal dimension values varied significantly within the leaf top and stalk interior groups (Kruskal–Wallis rank sum test; leaf top: *X*^2^ = 7.17, df = 2, and *p* value < 0.05; stalk interior: *X*^2^ = 10.90, df = 2, and *p* value < 0.01; Table S3). The *D*_f_ of the moderately degraded leaf tops is significantly higher than the mildly degraded leaf tops (Dunn’s test; *z* = −2.62 and *p* < 0.025), and the *D*_f_ of the severely degraded stalk interior was higher than the mildly and moderately degraded stalk interior groups (Dunn’s test; mild–severe: *z* = −2.79 and *p* < 0.01; moderate–severe: *z* = −2.92 and *p* < 0.01).

**Figure 6:**
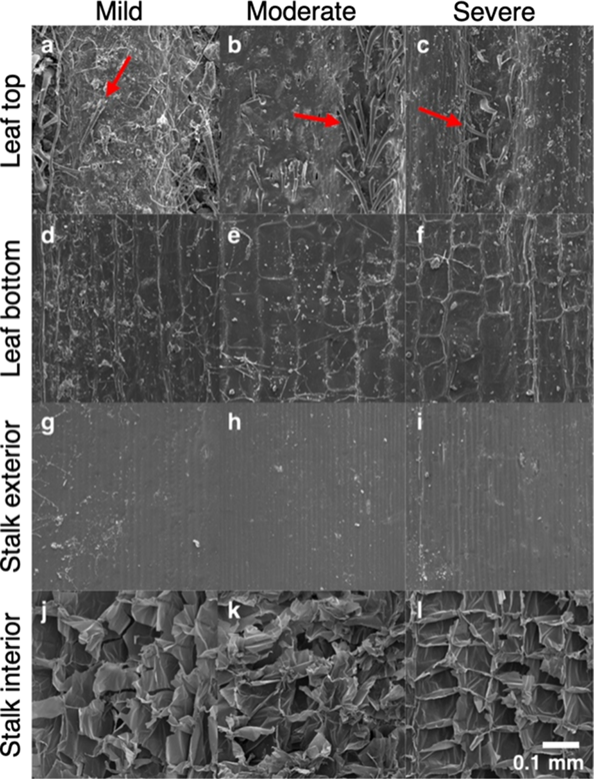
SEM micrographs showing the microscale texture of corn stover particle surfaces. The differences among the anatomical fractions are dramatic. The leaf top samples have a unique surface due to the presence of trichomes (red arrows). The differences among the variable biological degradation during storage are subtle at this scale. (a–c) Mildly, moderately, and severely degraded leaf top, respectively. (d–f) Mildly, moderately, and severely degraded leaf bottom, respectively. (g–i) Mildly, moderately, and severely degraded stalk exterior, respectively. (j–l) Mildly, moderately, and severely degraded stalk interior, respectively. Scale bar = 100 μm.

**Figure 7:**
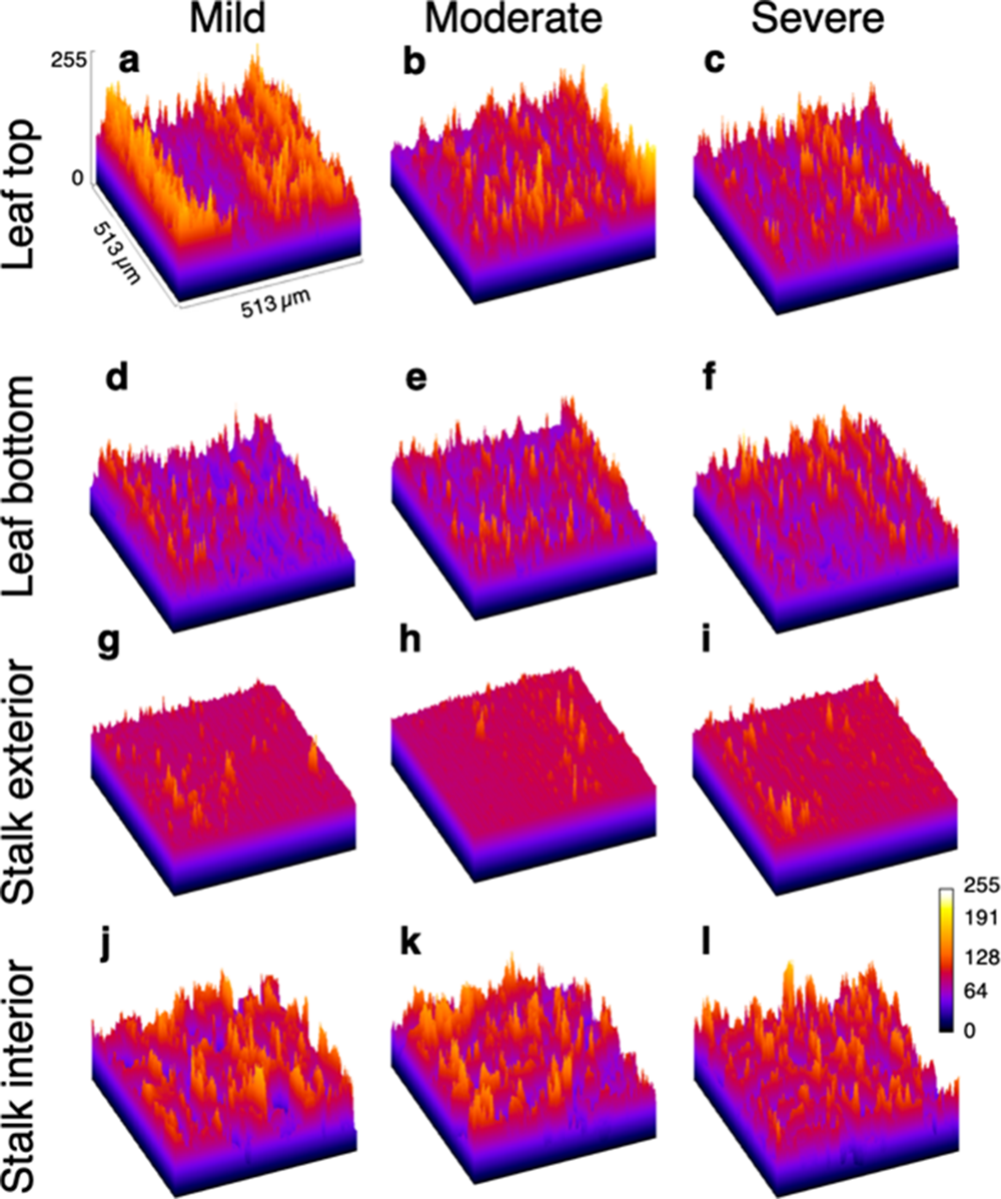
3D surface topology visualization calculated from SEM micrographs. Leaf top and stalk interior have the most varied surface topologies. Stalk exterior surfaces are relatively smooth. (a–c) Mildly, moderately, and severely degraded leaf top, respectively. (d–f) Mildly, moderately, and severely degraded leaf bottom, respectively. (g–i) mildly, moderately, and severely degraded stalk exterior, respectively. (j–l) Mildly, moderately, and severely degraded stalk interior, respectively.

The same basic approach to sampling, image capture, and analyses was applied to scanning electron microscopy (SEM) micrographs of the same materials. SEM micrographs were captured from three different regions of interest on 10 separate biomass pieces, and representative micrographs are shown in Figure 6. A moderate magnification was used to capture a region of interest hundreds of micrometers in dimension and was predicted to be a relevant scale for biomass conversion. Previous texture analysis of SEM micrographs of leaf surfaces found that the best correlation between surface adhesion and the fractal dimension analysis was from images captured at only 300× magnification.^39^ Some of the striking features visible at this scale include the cone-shaped trichomes on the leaf top surfaces, the smooth and uniform appearance of the stalk exterior, and the high topology created by the thin-walled parenchyma cells of the stalk interior surface. The 3D topology of the SEM micrographs is visualized in Figure 7.

**Figure 8:**
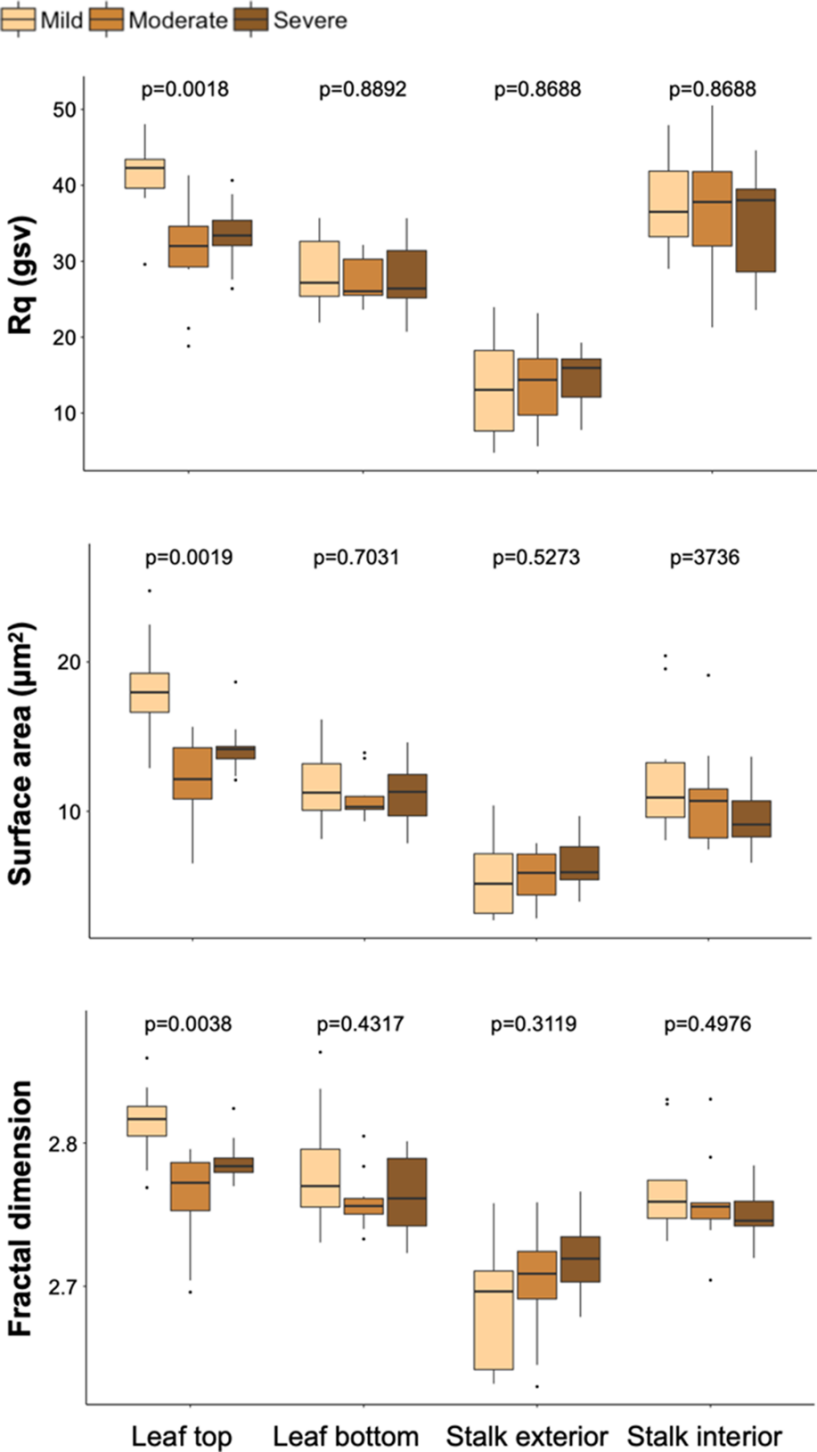
Surface texture quantification from SEM images. The Rq, topographical surface area, and fractal dimension were assessed. A Kruskal–Wallis test was used to assess significant differences among the varying levels of degradation for each anatomical fraction, and the Dunn test was used for post-hoc analysis. At this micrometer scale, increased biological degradation was not related with higher surface roughness or surface area.

Significant variation of the Rq value was only found for the leaf top surfaces (Kruskal–Wallis rank sum test; leaf top: *X*^2^ = 12.60, df = 2, and *p* value < 0.01). The mild group had a significantly higher Rq than both the other groups (Dunn’s test; mild–moderate: *z* = 3.28 and *p* < 0.01; mild–severe: *z* = 2.82 and *p* < 0.01).This may have been due to a higher observed amount of foreign material (possibly accumulated soil) present on this sample group (Figure 6e). Significant variation of topographical surface area was also only found in the leaf top (Kruskal–Wallis rank sum test; leaf top: *X*^2^ = 12.48, df = 2, and *p* value < 0.01; Table S4). The mildly degraded group had a significantly higher topographical surface area than the moderately degraded group (Dunn’s test; *z* = 3.51 and *p* < 0.001). Significant variation of fractal dimension was also found only in the leaf top surfaces (Kruskal–Wallis rank sum test; leaf top: *X*^2^ = 11.17, df = 2, and *p* value < 0.01). The mildly degraded sample group had a significantly higher *D*_f_ than the moderately degraded samples (Dunn’s test; *z* = 3.33 and *p* < 0.01). Overall, these results indicate that the level of degradation at the micrometer scale assessed by SEM is not associated with variability of surface roughness and surface area of the leaf bottom, stalk exterior, and stalk interior surfaces (Figure 8). Whether the level of degradation is the main cause of the variability of the leaf top surface is somewhat unclear due to the presence of an unidentified foreign material on the mild samples. One possibility is that this material may have microbial growth that was present only on the mildly degraded leaf top due to the presence of soluble sugars that had been previously utilized in the more degraded samples. This would indicate that a possible benefit to self-heating is a decreased chance of the foreign material being on the self-heated material because the conditions for growth are less favorable. The presence of this foreign material is an issue of working with realistic samples from the field.

### Total Surface Area and Porosity Measured Down to the Nanometer Scale Varied among Anatomical Fractions and Likely Have an Impact on Downstream Catalytic Conversion Processes

**Figure 9:**
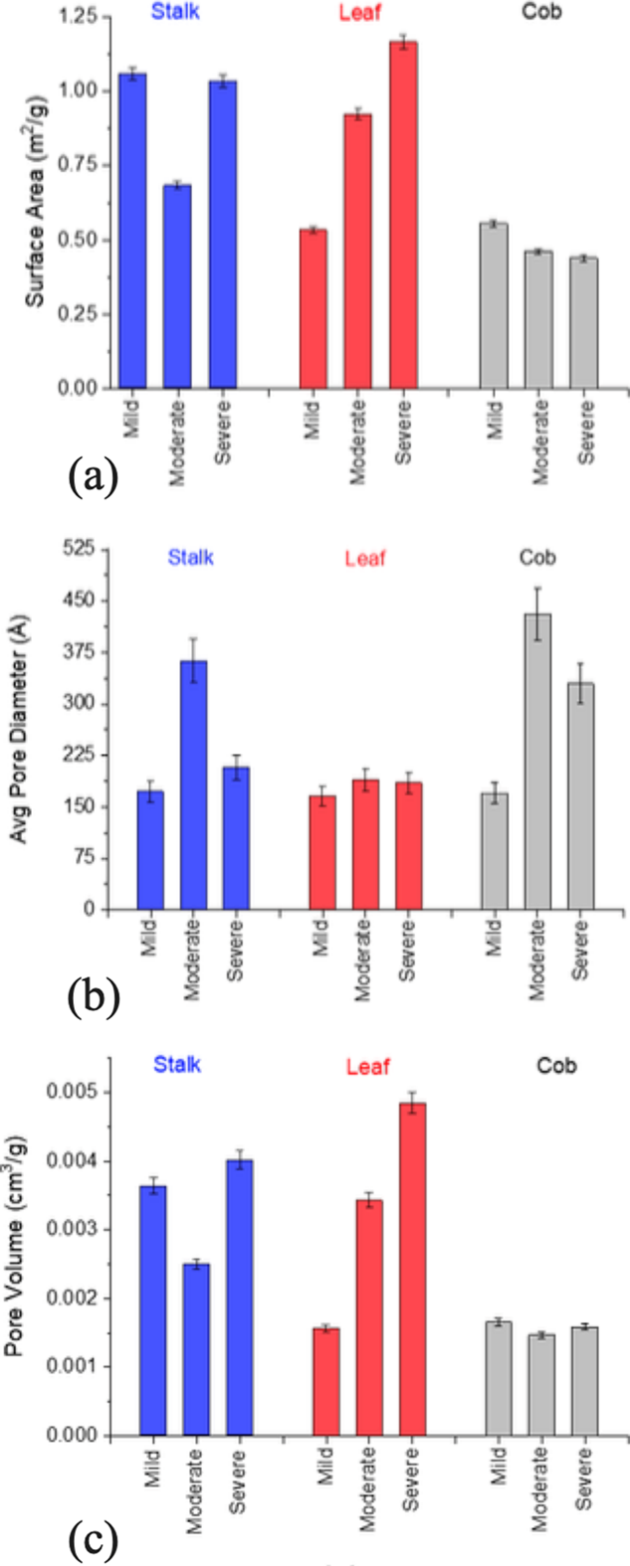
(a–c) Feedstock variability in (a) total surface area, (b) average pore diameter, and (c) total pore volume as a function of anatomical fraction (leaf, stalk, and cob) and the degree of biological heating (mild, moderate, and severe). Error bars represent one standard deviation from the mean.

Anatomical fractions vary in the surface area, porosity, and total pore volume (Figure 9). This variation is attributed to their form and biological function. Among the mildly heated sample measurements, the leaf and cob fraction had the lowest surface areas, while the stalk fraction produced the largest surface area—more than 2× the leaf surface area). Among the moderately heated sample measurements, the leaf fraction increased in both porosity and surface area with biological heating. The cob decreased in the surface area, and pore volume remained relatively unchanged. It is important to note that the cob surface was only a small portion of the ground material examined; the inner most or pith of the cob appeared to not be as affected by the biological heating and yet was the tissue fraction that dominated the volume of the analyzed cob sample. The moderately heated stalk sample (as compared to the mildly heated sample) decreased in the surface area accompanied by an increase in the average pore diameter and a decrease in the total pore volume. A possible mechanism that we are exploring further is the impact of lignin coalescence and migration. The most notable impacts on surface areas, pore diameters, and pore volumes were observed with the leaf fraction. The surface area and pore volume (i.e., porosity) monotonically increased with the degree of biological heating. In contrast, the average pore diameter remained relatively unaffected by the degree of biological heating. The cob decreased in the surface area and had a shift in pore size distribution. The stalk increased in the surface area and also increased in pore volume over diameters from 7.5 to 15 nm. In summary, changes in the surface area and porosity can be evaluated across a range of corn stover tissue types and degrees of degradation with nitrogen adsorption with the BET and NLDFT methods. It is important to note that the samples analyzed were all taken from the same bale of corn stover. However, even within a single bale, there is variability in the samples because they were assigned to the degree of biological heating categories based on the color and physical observation.

The surface area and porosity are highly impactful material properties for solid–liquid reactions and solid–gas reactions that directly influence the reaction rates of conversion processes (i.e., pretreatment and fermentation) and reaction selectivity (i.e., furfural and hydroxymethyl furfural). We have observed feedstock variability in the total surface area, total pore volume, and average pore diameters for three different anatomical fractions (leaf, stalk, and cob) with varying extents of biological heating (mild, moderate, and severe). The anatomical fractions that have undergone biological heating are expected to proceed via different reaction pathways and different reaction rates. The degradation rates not only depend on the chemical composition, density, surface area, and porosity of the anatomical fractions but also depend on the location in the bale, temperature gradient, oxygen supply, and time. Given the three degradation mechanisms (biological, thermal, and oxidative), we quantified physical (surface area, pore volume, and pore diameter) and chemical changes (i.e., surface energy) of the anatomical fractions exposed to varying degrees of biological heating (i.e., mild, moderate, and severe). Shown in Figure 9 are the results obtained for corn stalk, leaf, and cob that have been exposed to differing degrees of heating (mild, moderate, and severe). It is important to note that the initial classification of the degree of biological heating was based on visual classification; consequently, there are no specific details on the temperature, time, and oxic or anoxic microenvironments present within the bale of the exposed samples. In other words, a classification of moderate may be different for each of the anatomical fractions and perhaps within an anatomical fraction. The salient features of Figure 9 are as follows:

The surface area and total pore volume of the stalk were greater than the leaf and cob under mildly biologically heated conditions. The corn stalk surface area, average pore diameter, and total pore volume were all nonmonotonic as a function of the degree of biological heating. The stalk surface area decreased by 35% from mild biological heating to moderate biological heating—accompanied by a decrease in the total pore volume and an increase in the average pore diameter. Going from moderate to severe biological heating resulted in an increase in the surface area and total pore volume and a decrease in the average pore diameter for the corn stalk sample. Under mild biological heating conditions, the dramatic changes in the surface area, average pore diameter, and total pore volume can be attributed to the structural rearrangement of the surface and/or lignin migration/coalescence. As compared to the cob and leaf, the stalk was observed to be the most sensitive/reactive to conditions of biological heating. The initial reactivity of the stalk could be a direct consequence of having twice the porosity and twice the surface area as compared to the cob and leaf. The observations of nonmonotonic behavior may be illustrative of two or more competing mechanisms occurring during biological heating.

The leaf surface area, average pore diameter, and total pore volume all increased with increasing degree of biological heating. The surface area increased from around 0.5 m^2^/g (mild) to 1.2 m^2^/g (severe)—a 118% increase in the surface area. The most dramatic effect was in the total pore volume where an increase of 210% in the total pore volume was observed from the mild to severe biological heating. The average pore diameter remained relatively unchanged from mild to severe biological heating, indicating consistent and uniform biological heating effects for the leaf fraction.

The least impacted anatomical fraction was the cob. The cob surface area decreased by 21% [0.6 m^2^/g (mild) to 0.4 m^2^/g (severe)] with increasing severity of biological heating. The total pore volume remained relatively unchanged at around 0.0015 cm^3^/g. Pronounced effects were observed with the average pore diameter. The pore diameters for the mildly biologically heated samples (cob, stalk, and leaf) were all relatively close at around 15 nm. The average pore diameter of the moderately biologically heated cob increased by a factor of three as compared to the mildly biologically heated sample. The increase in pore diameters may be attributed to thermal events isolated to the cob surface. The decrease in the pore diameters going from moderate to severe may indicate a more severe surface treatment via increased temperature and/or partial oxidation.

Based on the surface area and total pore volume, the stalk is expected to be more reactive than the cob and leaf under identical reaction conditions (i.e., temperature and oxygen).^40^ Previous work has indicated that cobs and leaves are the most readily converted anatomical fractions by acid or alkaline pretreatment followed by saccharification and fermentation.^41,42^ These findings demonstrate that surface modifications that occur due to thermal and oxidative reactions during storage may alter reactivity to downstream hydrolysis and conversion and could potentially be adapted to enhance the reactivity of more recalcitrant fractions (like stalk). Heat and mass transport and bale location are all critical parameters in determining the effects of biological heating on the anatomical fractions.

### Surface Energy Is Affected by Biological Heating and Indicates Major Changes in the Chemical Structure of the Feedstock Surface

**Figure 10:**
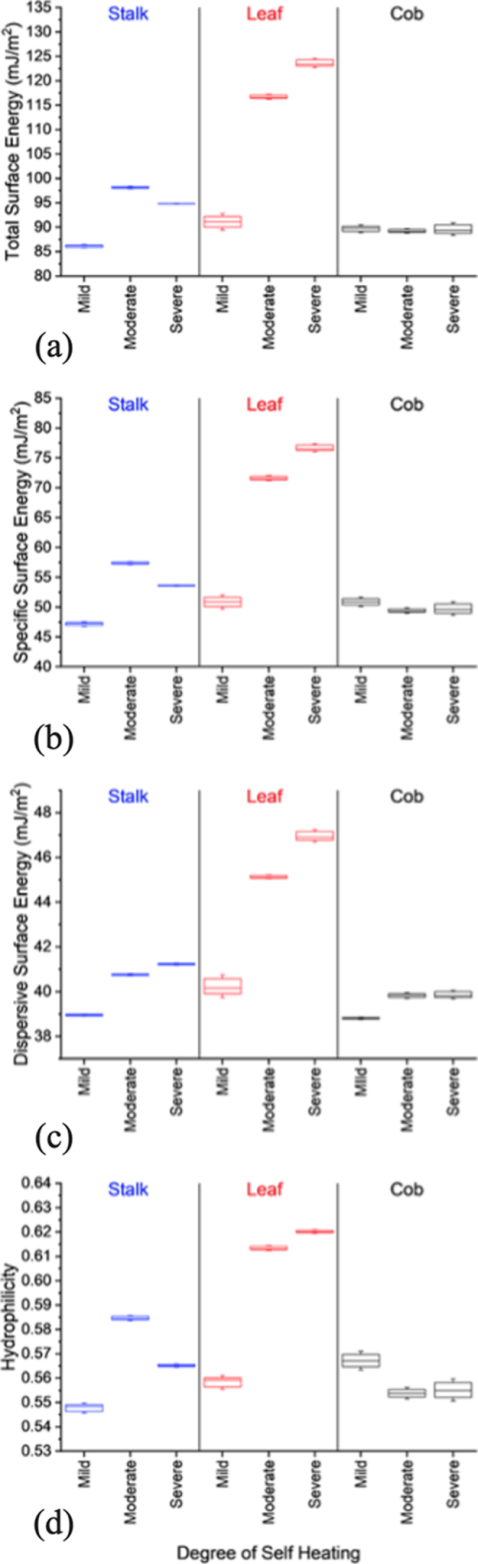
(a–d) Feedstock variability in (a) total surface energy, (b) dispersive surface energy, (c) specific surface energy (acid–base), and (d) hydrophilicity as a function of anatomical fraction (leaf, stalk, and cob) and the degree of biological heating (mild, moderate, and severe).

Surface energy characterization of corn stover anatomical fractions offers fundamental thermodynamic insights related to the intermolecular forces of van der Waals (dispersion force) and chemical (acid–base) bonds that give rise to several key properties of biomass particle surfaces including wettability, hydrophobicity, adhesion/cohesion, reactivity, and adsorption capacity. In other words, surface energy is a direct measure of the chemical environment of the surface and its comparative changes as function of processing (in our case, biological heating). Surface energy measurements were performed to quantify and track the changes in surface chemistry of anatomical fractions (cob, stalk, and leaf) as a function of biological heating. Shown in Figure 10 are the results of surface energy measurements performed on the anatomical fractions exposed to differing degrees of biological heating. The salient features of Figure 10 are as follows:

The total surface energy (Figure 10a), dispersive surface energy (Figure 10b), and specific surface energy (Figure 10c) of the mildly heated anatomical fractions are comparable in magnitude around 85–90, 39–40, and 47–51 mJ/m^2^, respectively. The total surface energy is the sum of the dispersive and specific components. Comparable surface energy values indicate that the surface chemistries of the three anatomical fractions are similar (if not identical) for the mildly biologically heated samples.

The total surface energy of the leaf fraction increased dramatically from the mildly heated (91 mJ/m^2^) to moderately heated (117 mJ/m^2^) to severely heated (124 mJ/m^2^), resulting in percentage increases of 28% from mild to moderate and 35% from mild to severe. The increase in the total surface energy is direct evidence of chemical modifications of the surface. A correlative study using the same corn stover biomass subjected to biological heating showed evidence for the chemical changes that happen in the cell wall. Using two-dimensional gas chromatography/mass spectrometry, they showed that hemicellulose and cellulose breakdown generated enhanced pyrolysis efficiency for the production of small oxygenates such as furfural, furanone, and pyranone derivatives.^26^

Although both the dispersive and specific surface energy components for the leaf fraction increase with the degree of biological heating, the largest contributor to the increase in the total surface energy is attributed to the specific surface energy component. The specific surface energy component increased from 51 mJ/m^2^ (mild) to 72 mJ/m^2^ (moderate) to 77 mJ/m^2^ (severe), resulting in a 40% increase from mild to moderate and a 51% increase from mild to severe. The dispersive surface energy component for the leaf fraction increased from 40 mJ/m^2^ (mild) to 45 mJ/m^2^ (moderate) to 47 mJ/m^2^ (severe). Of the three anatomical fractions tested, the leaf fraction demonstrated the largest change in surface energy, which is evidence that the leaf fraction is the most sensitive/reactive anatomical fraction under biological heating conditions.

**Figure 11:**
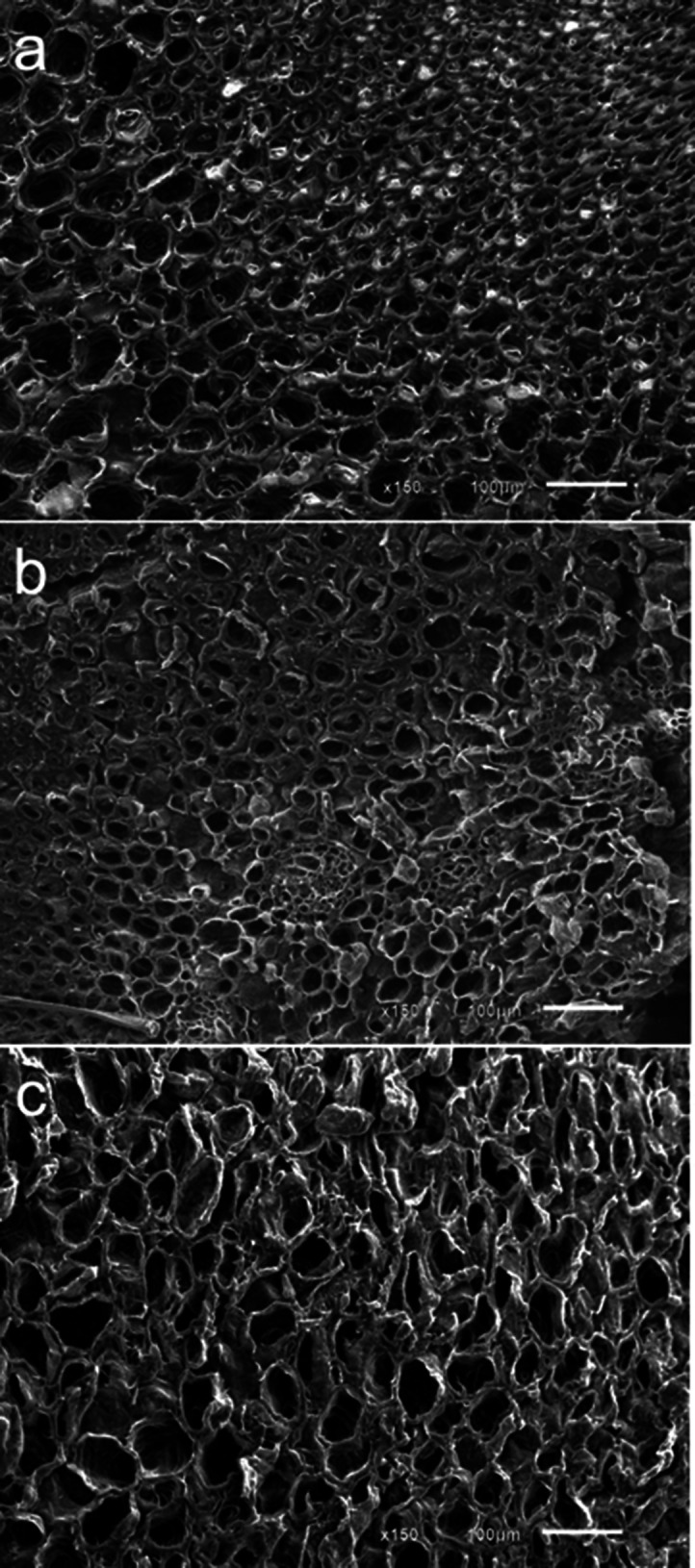
SEM micrographs of the corn stover cob internal structure (woody ring). (a, b) Morphologies of the woody ring tissue fraction with (a) mild or (b) moderate degradation maintain the cell structure and adhesion. (c) Severely degraded sample showing slight deformation of the cells and potential cell wall thinning.

The total surface energy for the cob remained relatively unchanged with respect to the degree of biological heating (89–90 mJ/m^2^). There was a slight decrease in the specific surface energy component (51 to 49 mJ/m^2^), accompanied by a slight increase in the dispersive component (39 to 40 mJ/m^2^). Because of the unique behavior of the cob material in terms of surface energy and the observation that the interior of the cob showed little evidence of biological degradation, we inspected the cob woody ring tissue by SEM. Previous work indicates that chaff, woody ring, and pith account for 21, 78, and 1 of corn cob on an oven-dried weight basis, respectively.^43^ Thus, woody ring tissue is a good representation of the cob samples for structural characterization and understanding of its response to biological degradation as well as its correlation to surface characteristics. The cob woody ring has a dense and well-organized lignified structure composed of small size parenchyma cells and vascular bundles. The morphologies of the woody ring under mild and moderate degradation are still intact without an obvious difference (Figure 11). The severely degraded sample shows slight structural distortion. These low structural variability results are in good agreement with the changes of pore volume, surface area, and surface energy measurements of cobs exposed to three levels of degradation, supporting that cob is the anatomical fraction least impacted by biological heating.

The stalk fraction demonstrated nonmonotonic behavior for the total surface energy [86 mJ/m^2^ (mild), 98 mJ/m^2^ (moderate), and 95 mJ/m^2^ (severe)] and the specific surface energy component [47 mJ/m^2^ (mild), 57 mJ/m^2^ (moderate), and 54 mJ/m^2^ (severe)], while the dispersive component increased with the degree of biological heating [39 mJ/m^2^ (mild) and 41 mJ/m^2^ (moderate/severe)]. The nonmonotonic behavior of the stalk fraction may illustrate competing degradation mechanisms as a function of the degree of biological heating. For example, the moderate biological heating may only include the effects of biological degradation and temperature effects, while in the severe biological heating case, the mechanism may be partial oxidation and thermal decomposition.

Under mild biological heating conditions, the cob is the most hydrophilic and the stalk is the least hydrophilic. Hydrophilicity is measure of the ability to absorb and retain water. Hydrophilicity values range from 0 to 1 where a value of 0 indicates extremely hydrophobic and a value of 1 indicates extremely hydrophilic. The hydrophilicity of the cob remains unchanged with the severity of biological heating (0.55–0.57). The hydrophilicity of the stalk is nonmonotonic with the degree of biological heating [0.55 (mild), 0.58 (moderate), and 0.56 (severe)]. Changes in the hydrophilicity correlate to the changes in the specific component of surface energy. These changes directly reflect the changes in surface chemistry of the surface carbonyl groups and hydroxyl groups.^26^ The leaf fraction monotonically increased in hydrophilicity with increasing degree of biological heating [0.56 (mild), 0.61 (moderate), and 0.62 (severe)]. Overall, there was a 10% increase in leaf hydrophilicity from the mild to severe biological heating.

## Conclusions

The surface textural analysis revealed that biological heating affects the surface roughness and surface area of corn stover tissues. At the millimeter scale, biological degradation is associated with increased surface roughness for the leaf and stalk interior surfaces. The surface texture of the stalk exterior and leaf bottom surfaces does not change. The surface area of the leaf top may increase with biological degradation, although this trend is not as clear. The surface area of the stalk interior is higher for the severely degraded group. At the micrometer scale, only the leaf top surfaces varied with the level of biological degradation. The mildly degraded leaf tops were the roughest and had the highest surface area, which may be due to the presence of the foreign material. At the nanometer scale, large changes were observed in the porosity, surface energy, and wettability of the anatomical fractions.

While biological degradation has been clearly shown to cause variable surface texture, the nature of this relationship is complex as it varies with the specific surface being compared, the scale, and the level of degradation. This study found that biological heating affects both the values of parameters calculated and how wide or narrow their distributions are. The millimeter scale is likely relevant to surfaces that will contact each other causing friction and effecting flowability, so the higher surface roughness of the leaf top and bottom and stalk interior surfaces at this scale may be undesirable. Increased surface roughness is also associated with increased hydrophobicity, which could pose a problem for the efficacy of pretreatments, although the increased surface area that corresponds to increases in surface roughness may offset this.

The effects of biological self-heating on anatomical corn stover fractions were observed to have pronounced impacts not only on the physical characteristics (i.e., surface area, pore volume/porosity, and pore diameter) but also on the fundamental thermodynamic surface properties (i.e., total dispersive and specific surface energies and wettability). The leaf fraction demonstrated the highest reactivity/sensitivity to biological self-heating conditions—evidenced by the large changes observed in the surface area, porosity, surface energy, and wettability. All increased with increasing degree of biological self-heating. The least reactive anatomical fraction was the cob, which remained relatively unchanged with respect to the degree of biological heating; the exception was the marked change in average pore diameter. The leaf fraction was the most hydrophilic or wettable fraction, in contrast to cob, which was the most hydrophobic fraction and the least wettable fraction indicating the likelihood for differing pretreatment and fermentation reaction rates based on the ability to absorb aqueous solutions. The observed differences in susceptibility to degradation across anatomical fractions can be viewed analogously to what can be expected with pretreatment and fermentation processes. This study revealed significant variations in surface attributes in anatomical fractions of corn stover as a function of biological heating and degradation in storage. These findings suggest that heterogeneity inherent to lignocellulosic feedstocks and variations across distinct plant fractions may confound standard approaches to bioprocessing. Fundamental understanding of surface properties and their variations across anatomical and tissue scales informs development of advanced fractionation technologies to improve feedstock handling and tune pretreatment chemistries to plant fractions with variable and multiscale factors of recalcitrance. Feedstock variability can be exploited to derive a value from “overlooked” fractions of lignocellulosic biomass through informed understanding of quality and thereby enable development of pathways tailored to end use applications for enhanced utilization and valorization.

## Supporting Information Available

Table S1, median and interquartile values of Rq, surface area, and fractal dimension calculated from stereomicrographs; Table S2, summary of results of the Kruskal–Wallis rank sum test on texture results from stereomicrographs; Table S3, median and interquartile values of Rq, surface area, and fractal dimension calculated from SEM micrographs; and Table S4, summary of results of the Kruskal–Wallis rank sum test on texture results from SEM micrographs (PDF)
