# Supplementary material for: Impacts of Biological Heating and Degradation during Bale Storage on the Surface Properties of Corn Stover
Source: ACS Sustain Chem Eng. 2020 Aug 13;8(37):13973–83. doi: 10.1021/acssuschemeng.0c03356 (PMC10906940; doi:10.1021/acssuschemeng.0c03356)
Supplement: Supplementary file 1 — sc0c03356_si_001.pdf [file sc0c03356_si_001.pdf]

Impacts of biological heating and degradation during bale storage on the surface properties of corn stover

Elizabeth Bose[1,‡], Juan H. Leal[2,‡], Amber N. Hoover[3], Yining Zeng[1], Chenlin Li[3], Allison E. Ray[3], Troy A. Semelsberger[2,*], and Bryon S. Donohoe[1,*]

[1]Bioenergy Center, National Renewable Energy Laboratory (NREL)

15013 Denver West Parkway, Golden, CO, 80401, USA;

[2]Material Physics Applications Division, Los Alamos National Laboratory,

P.O. Box 1663, Los Alamos, NM, 87545, USA;

[3]Energy & Environment Science & Technology, Idaho National Laboratory,

1955 N. Fremont Avenue, Idaho Falls, ID, 83415, USA

‡ Equal contributors

*Corresponding authors:

troy@lanl.gov

bryon.donohoe@nrel.gov

Keywords
biomass feedstock, surface characterization, corn stover, biological degradation, surface area, porosity

Supporting information

Pages S1-S4

Table S1. Median and interquartile values of Rq, surface area, and fractal dimension calculated from stereomicrographs.

Table S2. Summary of results of Kruskal-Wallis rank sum test on texture results from stereomicrographs.

Table S3. Median and interquartile values of Rq, surface area, and fractal dimension calculated from SEM micrographs.

Table S4. Summary of results of Kruskal-Wallis rank sum test on texture results from SEM micrographs.

**Table S1**		Median (IQR)		
Anatomical fraction	Level of degradation	Rq (gsv)	SA (mm²)	F_D
Leaf top	Mild	27.89 (25.74, 30.05)	1.69 (1.57, 1.81)	2.8114 (2.80215, 2.82065)
	Moderate	33.97 (29.53, 38.41)	1.94 (1.75, 2.13)	2.8238 (2.8050, 2.8426)
	Severe	31.94 (28.75, 35.12)	1.86 (1.72, 2)	2.8223 (2.8132, 2.8315)
Leaf bottom	Mild	26.84 (23.36, 30.31)	1.92 (1.73, 2.1)	2.8247 (2.8017, 2.8476)
	Moderate	24.93 (21.79, 28.07)	1.93 (1.78, 2.09)	2.8345 (2.8166, 2.8525)
	Severe	26.25 (22.32, 30.18)	1.96 (1.85, 2.07)	2.8239 (2.8028, 2.8450)
Stalk exterior	Mild	34.20 (29.64, 38.77)	2.02 (1.86, 2.18)	2.8415 (2.8306, 2.8524)
	Moderate	35.26 (32.56, 37.96)	2.18 (1.81, 2.56)	2.8444 (2.8270, 2.8617)
	Severe	29.17 (24.12, 34.21)	1.98 (1.71, 2.24)	2.8313 (2.8092, 2.8534)
Stalk interior	Mild	21.82 (19.91, 23.72)	1.65 (1.51, 1.8)	2.8121 (2.7823, 2.8418)
	Moderate	20.89 (18.75, 23.03)	1.72 (1.61, 1.83)	2.8172 (2.801, 2.8333)
	Severe	25.74 (23.22, 28.26)	2.09 (1.89, 2.29)	2.8386 (2.8219, 2.8552)

Table S1. Median and interquartile values of Rq, surface area, and fractal dimension calculated from stereomicrographs.

**Table S2**	Anatomical fraction	Kruskal-Wallis chi-squared	df	p-value
Rq	Leaf top	10.694	2	0.004762
	Leaf bottom	0.51871	2	0.7715
	Stalk exterior	4.1368	2	0.1264
	Stalk interior	16.114	2	0.0003169
SA	Leaf top	10.64	2	0.004893
	Leaf bottom	0.28903	2	0.8654
	Stalk exterior	4.3071	2	0.1161
	Stalk interior	19.355	2	6.27E-05

Db	Leaf top	7.1716	2	0.02771
	Leaf bottom	1.3548	2	0.5079
	Stalk exterior	3.5253	2	0.1716
	Stalk interior	10.903	2	0.004289

Table S2. Summary of results of Kruskal-Wallis rank sum test on texture results from stereomicrographs.

**Table S3**		Median (IQR)		
Anatomical fraction	Level of degradation	Rq (gsv)	SA (µm²)	F_D
Leaf top	Mild	42.28 (38.48, 46.09)	17.97 (15.35, 20.58)	2.8167 (2.796, 2.8374)
	Moderate	32 (26.65, 37.36)	12.15 (8.72, 15.59)	2.7723 (2.7387, 2.8058)
	Severe	33.39 (30.06, 36.72)	14.17 (13.34, 14.99)	2.7839 (2.7738, 2.7939)
Leaf bottom	Mild	27.17 (19.94, 34.4)	11.25 (8.13, 14.36)	2.77 (2.7296, 2.8104)
	Moderate	26.04 (21.29, 30.8)	10.29 (9.43, 11.16)	2.7561 (2.7452, 2.767)
	Severe	26.4 (20.16, 32.63)	11.3 (8.52, 14.07)	2.7614 (2.7143, 2.8085)
Stalk exterior	Mild	13.06 (2.45, 23.67)	5.15 (1.16, 9.14)	2.6964 (2.6276, 2.7652)
	Moderate	14.37 (6.95, 21.8)	5.88 (3.13, 8.62)	2.7088 (2.6756, 2.742)
	Severe	15.95 (10.91, 20.98)	5.91 (3.7, 8.12)	2.7193 (2.6877, 2.7508)
Stalk interior	Mild	36.5 (27.86, 45.15)	10.92 (7.27, 14.58)	2.7591 (2.7324, 2.7857)
	Moderate	37.79 (28, 47.59)	10.69 (7.41, 13.97)	2.7557 (2.7445, 2.7668)
	Severe	38.04 (27.14, 48.94)	9.12 (6.7, 11.53)	2.7458 (2.7285, 2.763)

Table S3. Median and interquartile values of Rq, surface area, and fractal dimension calculated from SEM micrographs.

**Table S4**	Anatomical fraction	Kruskal-Wallis chi-squared	df	p-value
Rq	Leaf top	12.596	2	0.00184
	Leaf bottom	0.23484	2	0.8892
	Stalk exterior	0.28129	2	0.8688

	Stalk interior	0.28129	2	0.8688
SA	Leaf top	12.48	2	0.00195
	Leaf bottom	0.70452	2	0.7031
	Stalk exterior	1.28	2	0.5273
	Stalk interior	1.969	2	0.3736
Db	Leaf top	11.166	2	0.00376
	Leaf bottom	1.68	2	0.4317
	Stalk exterior	2.3303	2	0.3119
	Stalk interior	1.3958	2	0.4976

Table S4. Summary of results of Kruskal-Wallis rank sum test on texture results from SEM micrographs.
